# Supplementary material for: Unveiling the Bioactive Potential of the Invasive Jellyfish Phyllorhiza punctata Through Integrative Transcriptomic and Proteomic Analyses
Source: Biomolecules. 2025 Aug 4;15(8):1121. doi: 10.3390/biom15081121 (PMC12383608; doi:10.3390/biom15081121)
Supplement: Supplementary file 1 [file biomolecules-15-01121-s001.zip › Suplementary Material/FileS3/PEPFOLD4/XP_065068582_14708/clusters.html]

 PEP-FOLD clusters


Download archive of all models  here   

|  |  |  |
| --- | --- | --- |
| # | file | sOPEP |
| # cluster 1 - Size: 32 |  |  |
|  | XP\_065068582\_14708-model1 | -21.796 |
|  | XP\_065068582\_14708-model1.1 | -21.6306 |
|  | XP\_065068582\_14708-model1.2 | -21.6061 |
|  | XP\_065068582\_14708-model1.3 | -21.5915 |
|  | XP\_065068582\_14708-model1.4 | -21.5648 |
|  | XP\_065068582\_14708-model1.5 | -21.5575 |
|  | XP\_065068582\_14708-model1.6 | -21.5575 |
|  | XP\_065068582\_14708-model1.7 | -21.5575 |
|  | XP\_065068582\_14708-model1.8 | -21.5273 |
|  | XP\_065068582\_14708-model1.9 | -21.5223 |
|  | XP\_065068582\_14708-model1.10 | -21.5223 |
|  | XP\_065068582\_14708-model1.11 | -21.5108 |
|  | XP\_065068582\_14708-model1.12 | -21.5044 |
|  | XP\_065068582\_14708-model1.13 | -21.5043 |
|  | XP\_065068582\_14708-model1.14 | -21.5043 |
|  | XP\_065068582\_14708-model1.15 | -21.4756 |
|  | XP\_065068582\_14708-model1.16 | -21.4469 |
|  | XP\_065068582\_14708-model1.17 | -21.4357 |
|  | XP\_065068582\_14708-model1.18 | -21.4334 |
|  | XP\_065068582\_14708-model1.19 | -21.4027 |
|  | XP\_065068582\_14708-model1.20 | -21.3985 |
|  | XP\_065068582\_14708-model1.21 | -21.3761 |
|  | XP\_065068582\_14708-model1.22 | -21.3712 |
|  | XP\_065068582\_14708-model1.23 | -21.3712 |
|  | XP\_065068582\_14708-model1.24 | -21.3697 |
|  | XP\_065068582\_14708-model1.25 | -21.3501 |
|  | XP\_065068582\_14708-model1.26 | -21.3499 |
|  | XP\_065068582\_14708-model1.27 | -21.3357 |
|  | XP\_065068582\_14708-model1.28 | -21.325 |
|  | XP\_065068582\_14708-model1.29 | -21.2511 |
|  | XP\_065068582\_14708-model1.30 | -21.1718 |
|  | XP\_065068582\_14708-model1.31 | -21.1718 |
| # cluster 2 - Size: 15 |  |  |
|  | XP\_065068582\_14708-model2 | -21.7746 |
|  | XP\_065068582\_14708-model2.1 | -21.5714 |
|  | XP\_065068582\_14708-model2.2 | -21.5325 |
|  | XP\_065068582\_14708-model2.3 | -21.5193 |
|  | XP\_065068582\_14708-model2.4 | -21.4146 |
|  | XP\_065068582\_14708-model2.5 | -21.3636 |
|  | XP\_065068582\_14708-model2.6 | -21.3377 |
|  | XP\_065068582\_14708-model2.7 | -21.3303 |
|  | XP\_065068582\_14708-model2.8 | -21.314 |
|  | XP\_065068582\_14708-model2.9 | -21.283 |
|  | XP\_065068582\_14708-model2.10 | -21.2543 |
|  | XP\_065068582\_14708-model2.11 | -21.2543 |
|  | XP\_065068582\_14708-model2.12 | -21.2543 |
|  | XP\_065068582\_14708-model2.13 | -21.2404 |
|  | XP\_065068582\_14708-model2.14 | -21.2356 |
| # cluster 3 - Size: 8 |  |  |
|  | XP\_065068582\_14708-model3 | -21.6745 |
|  | XP\_065068582\_14708-model3.1 | -21.6526 |
|  | XP\_065068582\_14708-model3.2 | -21.5726 |
|  | XP\_065068582\_14708-model3.3 | -21.5097 |
|  | XP\_065068582\_14708-model3.4 | -21.4531 |
|  | XP\_065068582\_14708-model3.5 | -21.4456 |
|  | XP\_065068582\_14708-model3.6 | -21.4039 |
|  | XP\_065068582\_14708-model3.7 | -21.3644 |
| # cluster 4 - Size: 4 |  |  |
|  | XP\_065068582\_14708-model4 | -21.5546 |
|  | XP\_065068582\_14708-model4.1 | -21.4831 |
|  | XP\_065068582\_14708-model4.2 | -21.2685 |
|  | XP\_065068582\_14708-model4.3 | -21.1162 |
| # cluster 5 - Size: 6 |  |  |
|  | XP\_065068582\_14708-model5 | -21.4321 |
|  | XP\_065068582\_14708-model5.1 | -21.4308 |
|  | XP\_065068582\_14708-model5.2 | -21.3136 |
|  | XP\_065068582\_14708-model5.3 | -21.3099 |
|  | XP\_065068582\_14708-model5.4 | -21.3099 |
|  | XP\_065068582\_14708-model5.5 | -21.3007 |
| # cluster 6 - Size: 7 |  |  |
|  | XP\_065068582\_14708-model6 | -21.4179 |
|  | XP\_065068582\_14708-model6.1 | -21.3958 |
|  | XP\_065068582\_14708-model6.2 | -21.3436 |
|  | XP\_065068582\_14708-model6.3 | -21.3282 |
|  | XP\_065068582\_14708-model6.4 | -21.2499 |
|  | XP\_065068582\_14708-model6.5 | -21.2037 |
|  | XP\_065068582\_14708-model6.6 | -21.1957 |
| # cluster 7 - Size: 13 |  |  |
|  | XP\_065068582\_14708-model7 | -21.4064 |
|  | XP\_065068582\_14708-model7.1 | -21.3241 |
|  | XP\_065068582\_14708-model7.2 | -21.3026 |
|  | XP\_065068582\_14708-model7.3 | -21.285 |
|  | XP\_065068582\_14708-model7.4 | -21.2668 |
|  | XP\_065068582\_14708-model7.5 | -21.2376 |
|  | XP\_065068582\_14708-model7.6 | -21.2145 |
|  | XP\_065068582\_14708-model7.7 | -21.204 |
|  | XP\_065068582\_14708-model7.8 | -21.1358 |
|  | XP\_065068582\_14708-model7.9 | -21.1264 |
|  | XP\_065068582\_14708-model7.10 | -21.1118 |
|  | XP\_065068582\_14708-model7.11 | -21.1058 |
|  | XP\_065068582\_14708-model7.12 | -21.0958 |
| # cluster 8 - Size: 4 |  |  |
|  | XP\_065068582\_14708-model8 | -21.3927 |
|  | XP\_065068582\_14708-model8.1 | -21.3418 |
|  | XP\_065068582\_14708-model8.2 | -21.3418 |
|  | XP\_065068582\_14708-model8.3 | -21.3164 |
| # cluster 9 - Size: 7 |  |  |
|  | XP\_065068582\_14708-model9 | -21.3749 |
|  | XP\_065068582\_14708-model9.1 | -21.3675 |
|  | XP\_065068582\_14708-model9.2 | -21.3383 |
|  | XP\_065068582\_14708-model9.3 | -21.3383 |
|  | XP\_065068582\_14708-model9.4 | -21.2997 |
|  | XP\_065068582\_14708-model9.5 | -21.2997 |
|  | XP\_065068582\_14708-model9.6 | -21.2284 |
| # cluster 10 - Size: 4 |  |  |
|  | XP\_065068582\_14708-model10 | -21.3295 |
|  | XP\_065068582\_14708-model10.1 | -21.3251 |
|  | XP\_065068582\_14708-model10.2 | -21.2401 |
|  | XP\_065068582\_14708-model10.3 | -21.2378 |
|  |  |  |
